# Postoperative amnesia in a patient undergoing general anesthesia for electro-physiologic (EP) catheter ablation of an irritable atrial focus

**Published:** 2014-10-31

**Authors:** Aris Sophocles, Linda Chen, David Lin, Renyu Liu

**Affiliations:** 1 Department of Anesthesiology and Critical Care, Perelman School of Medicine at the University of Pennsylvania; 2 Penn Heart and Vascular Center, Perelman School of Medicine at the University of Pennsylvania

## Abstract

This case report describes the anesthetic management of a 67-year-old who underwent a catheter based pulmonary vein isolation (PVI) of long-standing, persistent atrial fibrillation. When the patient awoke from the 6.5 hour procedure, he was found to have a transient retrograde and anterograde amnesia that persisted for 18–24 hours postoperatively. This is a unique instance of global amnesia following a cardiac ablation procedure under prolonged general anesthesia. This case study highlights important topics in postoperative cognitive deficits including the differential diagnosis, risk factors, and strategies for optimizing patient outcomes in high risk procedures.

## Introduction

This case involves a 67 year old man who underwent a catheter based pulmonary vein isolation of long-standing, persistent atrial fibrillation under general anesthesia. The procedure was successful in restoring his sinus rhythm. However, upon awakening from anesthesia he had significant amnesia including the inability to remember events preceding the procedure and orientation only to self for the initial 18 hours after emerging from anesthesia. In this case report we describe the procedural and anesthetic risk factors for, and management of, postoperative cognigitve deficits in the context of this patient’s unusual history and presentation. Permission to publish this case reprot was obtained from the Institutional Review Board (IRB) of the Hospital of the University of Pennsylvania. This paper passed plagiarism detection using iThenticate (http://www.ithenticate.com/, accessed date April 22, 2013)

## Case Description

A 67-year-old patient (ASA-PS-3; 70cm; 81kg) with a past medical history significant for anxiety, hyperlipidemia, benign prostatic hypertrophy, and medically refractory atrial fibrillation underwent catheter ablation of the pulmonary veins under general anesthesia. He had suffered from intermittent atrial fibrillation refractory to medical management for several years that over the preceding months became persistent. His home medication regimen included metoprolol, flecainide, aspirin, warfarin, fish oil, pravachol, and a multivitamin. After premedication with intravenous (IV) midazolam 2 mg, propofol 100 mg was administered for induction of general anesthesia. The airway was secured with an endotracheal tube uneventfully. Preoperative antibiotics included cefazolin 1g IV. The anesthetic course consisted of two portions. The patient was initially maintained on endtidal sevoflurane 1–2% with volume control ventilation. An hour into the procedure the anesthetic regimen was switched to total intravenous anesthesia (TIVA) with jet ventilation, a technique that had previously been shown to minimize diphragmatic movement and improve visualization and catheter stability for electrophysiologic catheter ablation procedures.[1] [is there a reason for the changeover specifically at the 1 hour marker or was the timing arbitrary?] Sevoflurane was discontinued and TIVA was started with propofol 60 mcg/kg/hr and remifentanyl 0.16 mcg/kg/hr. The patient’s blood pressure was stable throughout the case with systolic pressures ranging from 100–120 mmHg and diastolic pressures 50–70 mmHg. His O_2_ saturation remained between 96–100% with a fraction of inspired oxygen between 0.5–0.7. The end-tidal CO_2_ was kept between 30 and 40 mmHg. Temperature was measured via a nasal probe and was kept between 36–37 degrees Celsius. The duration of the procedure was 6.5 hours. Intraoperative arterial blood gasses (ABGs) were within normal limits and stable when checked at two, three, and four hours into the case. The initial arterial blood gas was as follows: pH 7.37, PCO_2_ 45 mmHg, PO_2_ 469 mmHg, base excess 0.5 mEq/liter, bicarbonate 24.5 mEq/liter, O_2_ saturation 100%. Subsequent arterial blood gases were stable and within normal limits. Postoperative complete blood counts and chemistry panels were not significantly different from preoperative values and were within normal limits. Over the 6.5 hour procedure, the total propofol and remifentanil doses were 2,310 mg and 3.62 mg, respectively.

At the conclusion of the procedure, the anesthetic agents were all discontinued and the patient was extubated without complications in the EP lab. The patient was fully alert and able to follow commands, but was unable to recall what procedure he had done, recognize his cardiologist or anesthesiologist, or recall events from the night before his surgery including how he arrived at the hospital. He did know his name and date of birth. His affect was calm and was otherwise appropriate. A full neurologic assessment was performed by the neurologist and cranial nerves II-XII were intact, strength was appropriate and equal bilaterally, and he did not have any gait difficulties, vision changes, dysarthria, or dysphagia. His electrolytes, glucose, and vital signs were all stable and within normal limits. A head computerized tomography 2 hours post-op was negative for acute hemorrhage, mass effect, hydrocephalus, or evidence of cerebral infarct. The consulting neurologist did not find any other focal deficits, and did not think that the global amnesia was from a cerebrovascular accident.

About 18 hours post-procedure he began to show improvement, knowing where he was, recognizing his wife, and being able to vaguely recall that he had a problem with atrial fibrillation. By 24 hours post-procedure he was alert and oriented to person, place, and time, was conversing appropriately, could recount events prior to his procedure, and remembered feeling confused immediately after his procedure.

## Discussion

The differential diagnosis for this case includes a cerebrovascular accident (CVA), residual effects of anesthesia, metabolic derangements, and inadequate intraoperative oxygenation or ventilation. In general, left heart procedures can have up to a 2–7% risk of embolic complications. However, the patient was anticoagulated with intravenous heparin for the entirety of the procedure with activated clotting times in the 350 second range making an embolic event less likely. Metabolic derangement or intaoperative hypoxia or hypercarbia are unlikely given the patient’s normal perioperative laboratory values and vital signs. A CVA from micoremboli is possible despite the negative head omputerized tomography given its low sensitivity for detecting microemboli and that a MRI, which is more sensitive, was not preformed. The neurologist did not believe the amnesia was from a CVA but rather from residual effects of anesthesia. However, all of the medications used in this case were short acting, therefore making it unlikly that such prolonged amnesia would be due to the residual pharmacological effects of anesthesia. The etiology of the amnesia in this case is likely multifactorial which is consistent with a recent study on transient global amenesia suggesting that the exact etiology remains unknown and may be multifactorial.[2]

Was this a case of postoperative cognitive dysfunction (POCD)? POCD requries a battery of preoperative and postoperative neuropsychological tests[3]. The term has been used to study patient and procedural risk factors for the development of postoperative cognitive deficits. Common symptoms of POCD are memory deficits and a reduced abilty to handle intellectual challenges. It most typically affects domains of learning and memory, executive function, and processing speed.[3] This case of postoperative global amnesia cannot be classified as POCD because no comprehensive pre-operative or post-operative neuropsychological tests were performed. Furthermore, the clinical manifestation of amnesia resolved within 24 hrs. While this was not a case of POCD, the literature on POCDcan be of help in understanding this patient’s risk factors for post-operative cognitive complications, and what can be done to minimize these risks.

Risk factors for POCD can be divided into pre-operative, operative, and post-operative. Pre-operative risk factors include advanced age, pre-existing neurologic disease, polypharmacy, alcohol or sedative-hypnotic withdrawal, endocrine dysfunction, sepsis, depression, dementia and anxiety.[4] Operative risk factors include cerebral hypoperfusion, hypoxia, hypercarbia, air embolus and micro-emboli in cardiac surgery, anticholinergic drugs, and benzodiazepines. The most significant post-operative risk factors include abnormal surroundings, disruptions in sleep cycles and daily routines, and diminished autonomy.[5,6]

Potential contributing factors to this patient’s transient amnesia include his age, history of anxiety, peri-operative use of a benzodiazepine, unfamiliar surroundings, and the length of the ablation procedure including the prolonged exposure to anesthetic agents.[7] Though use of longer acting benzodiazepines like diazepam has been associated with POCD, premedication with midazolam has been studied and has been shown not to be a risk factor for POCD.[8,9] A recent study of patients randomly assigned to propafol or sevoflurane did not find a difference in the rate of POCD between the two agents.[9–11] Intra-operative cerebral hypoperfusion, hypoxia, and hypercarbia are not likely contributing factors given the patient’s hemodynamic stability and normal ABGs throughout the case. The inflammatory response from major surgeries and embolic events from orthopedic surgeries have been identified as contributing factors to POCD. The effect of EP cardiac procedures on the incidence of POCD has not yet been studied.[5]A well-established, independent risk factor for POCD is age, with people 60 years and older having increased incidence of POCD post-operatively. Moller, et al. conducted one of the larger studies that examined 1218 patients aged 60 years and older with neuropsychological tests before, 1 week after, and 3 months after surgery. The study found POCD present in 25.8 % of patients 1 week after surgery and 9.9% 3 months after surgery compared to 3.4 % and 2.8% of age matched hospitalized patients not undergoing surgery.[12] The only statistically significant risk factor for long-term POCD was age. Supporting age as a predictor for POCD is a meta-analysis of 18 studies by Cryns, et al. that found surgery to have a significant impact on long-term POCD in the elderly.[13]

Minimizing the risk of POCD begins with pre-operative assessment, carefully assessing the patient’s physical and mental status, looking for signs of perceptual and cognitive deficits, and looking for potential drug interaction between the anesthetic plan and the patient’s medications. In this case the patient’s risk factors was his age >60, history of anxiety, the prolonged duration of the ablation procedure, and increased risk of micro emboli from the EP procedure.[14] Perioperative considerations include maintaining cerebral oxygenation, hemodynamic stability, minimizing the surgical stress response, and monitoring for electrolyte abnormalities, all of which were done for this patient. General pharmacokinetic and pharmacodynamic approaches in the elderly include starting low and going slow, choosing medications with shorter half-lives rather than longer half-lives, and minimizing the number of medications. Recent evidence suggest that minimizing the duration and exposure to anesthetic agents may improve post-operative cognitive function.[7] Post-operatively, maintaining adequate analgesia, normal sleep wake cycles, and frequent orientation will help minimize POCD. Management for POCD begins with ruling out all organic and reversible etiologies including electrolyte imbalance, hypoglycemia, sepsis, shock, hypercarbia, hypoxia, and stroke. For symptom management of acute delirium with agitation, oral haloperidol is the preferred treatment and is ideally administered at night before sleep.

Attributing this patient’s amnesia to his age, history of anxiety, and abnormal surroundings is likely only a partial explanation. Whether there is a correlation between lengthy cardiac EP procedures and postoperative cogntive complications requires further studies. EP procedures can often last several hours requiring prolonged exposure to anesthetic agents, and thromboembolic events are a known potential complication of these procedures. A 2011 prospective trial looking at micro-embolization during ablation of atrial fibrillation found MRI evidence of postoperative acute cerebral lesions in 8.5% of patients. [15] Stroke complications from radiofrequency ablation of atrial fibrillation have been reported to be as high as 5% in patients >60 years old.[16] A recent large radominzed control trial supported the use of intra-operative BiSpectral index and cerebral oxygen saturation monitoring to reduce the incidence of POCD in high-risk patients by minimizing exposure to anesthetic agents.[7,14] Given the risk factors for POCD with catheter ablation of AF, including patient age, risk of thormboembolic events, and prolonged exposure to anesthetic agents, it would be informative to know how each of these risk factors contributes to the incidence of postoperative cogntive deficits with this procedure.
